# The Impact of Insecticides on Mycelial Growth of *Metarhizium* spp. and Their Efficacy in Controlling Larvae and Pupae of the House Fly (*Musca domestica* L.)

**DOI:** 10.21315/tlsr2025.36.2.6

**Published:** 2025-07-31

**Authors:** Duanpen Wongsorn, Benya Saenmahayak, Nittaya Pitiwittayakul, Surachai Rattanasuk

**Affiliations:** 1 Department of Plant Science, Faculty of Agricultural Innovation and Technology, Rajamangala University of Technology Isan, Nakhon Ratchasima 30000, Thailand; 2 Department of Animal Science, Faculty of Agricultural Innovation and Technology, Rajamangala University of Technology Isan, Nakhon Ratchasima 30000, Thailand; 3 Major of Biology, Department of Science and Technology, Faculty of Liberal Arts and Science, Roi Et Rajabhat University, Roi Et 45120, Thailand

**Keywords:** Entomopathogenic Fungi, Biological Control, Pest Management, Pyrethroids

## Abstract

*Metarhizium* spp. are entomopathogenic hyphomycete fungi with great potential as biological control agents against insects and as a component within integrated pest management systems. This study evaluated 10 *Metarhizium* spp. isolates for their effectiveness against house fly larvae and pupae. The isolates with the highest infection rates were tested for compatibility with insecticides. NM*Met*_SS9/2 and NM*Met*_CLPK4/1 were the most effective, with infection rates of 86.67% and 60.00% for larvae and pupae, respectively. Both isolates were cultured on Potato Dextrose Agar (PDA) mixed with Cypas^®^ 250 EC (100 mL and 50 mL per 6.25 L) and Tanidil^®^-T (100 g/L and 50 g/L) to assess the impact of insecticides on mycelial growth. After 28 days, the radial growth of NM*Met*_SS9/2 (81.33 mm) and NM*Met*_CLPK4/1 (77.67 mm) on PDA with Cypas^®^ 250 EC (50 mL per 6.25 L) showed no significant differences (*p* > 0.05) compared to growth on PDA alone. A spore suspension (1 × 10^8^ spores/mL) of NM*Met*_SS9/2 and NM*Met*_CLPK4/1 cultured on PDA with Cypas^®^ 250 EC (50 mL per 6.25 L) was used to control house fly larvae and pupae, resulting in mortality rates of 93.33% (NM*Met*_SS9/2) and 75.56% (NM*Met*_CLPK4/1), with infection rates of 77.78% and 71.11%, respectively. No significant differences (*p* > 0.05) were observed in mortality or infection rates compared to spores cultured on PDA without insecticides. These findings confirm that NM*Met*_SS9/2 and NM*Met*_CLPK4/1 are highly effective against house fly larvae and pupae and can proliferate on media containing Cypas^®^ 250 EC at 50 mL per 6.25 L without compromising their insecticidal properties, making them promising candidates for integrated pest management strategies.

**Highlights**

- The NM*Met*_SS9/2 and NM*Met*_CLPK4/1 isolates exhibited the highest infection rates for larvae and pupae, respectively.
- Cypas^®^ 250 EC was less toxic to the hyphal growth of the NM*Met*_SS9/2 and NM*Met*_CLPK4/1 isolates than Tanidil^®^-T.
- The NM*Met*_SS9/2 and NM*Met*_CLPK4/1 isolates cultured on PDA with Cypas^®^ 250 EC demonstrated house fly control effectiveness comparable to those grown on PDA alone.

## INTRODUCTION

The house fly, scientifically known as *Musca domestica* L. (Diptera: Muscidae), is a pest that carries a multitude of pathogens, which affect both humans and animals worldwide. Houseflies serve as vectors for the mechanical transmission of a wide range of pathogens, including helminthic eggs, protozoan cysts and trophozoites, bacteria, fungi and viruses, which they disseminate through their vomit or excreta. These insects are implicated in the spread of over 100 types of pathogens, such as those responsible for cholera, anthrax, Shigella infections, ORF virus and ascariasis (Olagunju 2022). It is typically found in residential areas, waste disposal sites and locations with decaying food or animal remains (Abbas *et al.* 2013).

House flies can produce more than two broods or generations per year (multivoltine insects). They undergo 10–12 life cycles annually in temperate climates, with their populations peaking during the summer months. These flies neither migrate seasonally, enter diapause, nor survive under adverse environmental conditions. Consequently, their populations align periods of active development and reproduction with seasons when resources are most abundant (Reynolds 2017). Furthermore, the house fly exhibits a high rate of reproduction. It lays approximately 120 to 150 eggs per batch and can lay eggs 5 to 20 times, resulting in an average of 500 eggs over its lifetime (Iqbal *et al.* 2014). This rapid reproduction contributes to its status as a significant pest.

Chemical agents are frequently utilised to address house fly infestations due to their convenience and swift control capabilities. Organophosphates, carbamates and organochlorines are selected for their immediate effectiveness. Organophosphate insecticides, which are esters of phosphoric acid or its sulfur analogs, are extensively utilised for controlling insect pests due to their potent toxicity, rapid environmental degradation and selective biological activity. Their mechanism of action involves inhibiting acetylcholinesterase (AChE), an enzyme responsible for breaking down acetylcholine, thereby disrupting neural transmission, inducing hyperexcitation and ultimately resulting in the death of the target organisms (Barata *et al.* 2004). Pyrethroid insecticides are the most commonly utilised class for controlling house flies due to their high efficacy, low mammalian toxicity, brief environmental persistence and limited impact on nontarget organisms (Elliott 1980).

However, these chemicals can have detrimental effects on users and may linger in produce and the environment. Moreover, insects can develop resistance to these chemicals (Aktar *et al*. 2009; Abbasi *et al.* 2023). Acevedo *et al.* (2009) observed that house flies developed resistance against two commercial insecticides, namely 2,2-dicholovinyl dimethyl phosphate (DDVP) and permethrin. Additionally, house flies also developed resistance to permethrin, deltamethrin, beta-cypermethrin and propoxur (Wang *et al*. 2019).

To achieve effective insect control, alternatives such as botanical extracts and various bacterial strains are being investigated as potential replacements or supplements to chemical insecticides. This is in line with the Integrated Pest Management (IPM) approach (Miana *et al.* 2018). Significantly, entomopathogenic fungi, especially *Metarhizium* spp., are viewed as a promising solution for integrated pest management. This is due to their limited host range, safety, environmental compatibility and the feasibility of mass production. These fungi can target a wide range of insect orders, including Lepidoptera, Hymenoptera, Hemiptera, Homoptera, Heteroptera, Diptera and Coleoptera (Balachander *et al.* 2009; Saranraj & Jayaprakash 2017).

However, challenges emerge when insecticides are combined with entomopathogenic fungi or when fungi are used in areas previously treated with insecticides. Certain chemicals can adversely affect the growth, vitality and efficiency of fungi. For instance, specific chemical insecticides, such as Profenofos (50 EC), Indoxacarb (14.5% EC) and Methyldemeton, significantly impact fungal growth, while others, like Chlorpyriphos 20 EC, have a milder effect (Amutha *et al.* 2010). Moreover, the use of insecticides at rates divergent from the recommended label rates can also influence fungi (Abidin *et al.* 2017; Pelizza *et al.* 2018). Consequently, it is crucial to study the impact of commonly used insecticides on the efficacy of entomopathogenic fungi in controlling house flies for the development of effective Integrated Pest Management strategies in the future.

This research aimed to study the effects of insecticide on the growth of mycelium of *Metarhizium* spp. and to test the efficacy of *Metarhizium* spp. cultured on media mixed with insecticide in controlling larvae and pupae of the house fly.

## MATERIALS AND METHODS

### The Effective Screening of *Metarhizium* spp. for Controlling House Flies

#### The fungal spore preparation

The 10 *Metarhizium* spp. isolates (NM*Met*_DKT9/1, NM*Met*_SN2/1, NM*Met*_CLPK4/1, NM*Met*_NBM10/1, NM*Met*_LTMC7/2, NM*Met*_KTLS7/3, NM*Met*_BAL7/1, NM*Met*_NS7/2, NM*Met*_SS9/2 and NM*Met*_SS10/3) obtained from plant protection laboratory, Department of Plant Science, RMUTI Thailand were cultured on Potato Dextrose Agar (PDA). The culture was maintained in darkness at a temperature of 28°C–30°C for 21 days. Subsequently, the spores were harvested using a surfactant (Tween 20^®^, 0.05%). The fungal culture was diluted in a petri dish, and a sterilised loop was employed to scrape the mycelium and spores, which were then filtered through sterile, double-layered muslin cloth to remove solid debris. The spore suspension was then quantified using a Hemocytometer under a microscope at 40× magnification. The concentration of the spore suspension was adjusted to a density of 1 × 10^8^ spores/mL. The fungal spore preparation method was modified from Bharathi *et al.* (2022).

#### Insect culture

Adult house flies were sourced from a poultry farm located at the training center of Nong Rawieng, RMUTI, in Nakhon Ratchasima, Thailand (latitude 14°57’42.5”N and longitude 102°10’19.6”E). Sampling methods included net collection and baited traps, and the collection was conducted between 09:00 a.m. and 12:00 p.m. The collected house flies were subsequently classified following the criteria outlined by Geden *et al.* (2021). They were then reared in mesh cages with dimensions of 30 cm × 30 cm × 30 cm. The adult house flies were nourished with a synthetic diet comprising powdered milk (25 g) and dry yeast (25 g) and sugar (50 g). Upon laying eggs, the resulting larvae were fed a semi-artificial diet consisting of 50 g of coarse bran, 10 g of fine bran and 1 g of pineapple fruit. These ingredients were thoroughly mixed using a blender (Ali *et al*. 2024). The rearing process was conducted in a laboratory setting, maintaining a temperature of 25°C–30°C, following the methodologies prescribed by Mishra *et al.* (2011). Once the larvae reached the third instar (30 h–32 h after hatching), one-day-old pupae (24 h after pupation) were prepared for subsequent testing.

#### House fly assay

The third instar larvae or one-day-old pupae of the house fly were placed in 20-ounce plastic cups containing a semi-artificial diet mixed with suspended spores of *Metarhizium* spp., with each isolate at a final concentration of 1 × 10^8^ spores/mL. The treatments were compared with two controls: A semi-artificial diet without any additives and a semi-artificial diet containing Tween 20^®^ (0.05%). The experiment was conducted using a Completely Randomised Design (CRD) with three replicates for each treatment. Each replicate (plastic cup) contained 30 house fly larvae or pupae.

After inoculating the *Metarhizium* spp. isolates on larvae or pupae, they were transferred to an incubator in the dark at a temperature of 28°C–30°C and 60%–80% relative humidity (R.H.). When dead larvae and pupae were found, the insects’ skin surface was treated with a 0.5% Sodium Hypochlorite solution for 3 to 5 min, followed by rinsing with sterile water for 3 to 5 min, repeated twice. The larvae and pupae were then transferred to a moist chamber and incubated in the dark at a temperature of 28°C–30°C and 60%–80% R.H. Daily observations of mortality and fungal infection were recorded for 14 consecutive days. Correction for mortality in the control treatment were done using Abbott’s formula (Abbott 1925) as following;


[(Test mortality-Control mortality)×100]/[100-Control mortality]

### The Mycelial Growth of *Metarhizium* spp. on the Culture Medium Mixed with Insecticides

#### Culture media preparation

The culture medium for the fungal culture was prepared using Potato Dextrose Agar (PDA). Initially, 200 g of potato were boiled in water for 15 to 20 min until the boiling point was reached. The mixture was subsequently filtered to retain only the water. Various additives were then incorporated into the mixture, including 20 g of glucose and 20 g of agar. After autoclaving (at 121°C for 15 min) and cooling down the media, the insecticides (Cypas^®^ 250 EC. and Tanidil^®^-T, as shown in Table 1) were added at two concentrations: the recommended application rate and half the recommended rate. For Cypas^®^ 250 EC, the concentrations were 100 mL and 50 mL per 6.25 L, and for Tanidil^®^-T, they were 100 g/L and 50 g/L. The mixture was then shaken for 2 min to achieve a homogeneous distribution of the added compounds. This method was modified from Schumacher and Poehling (2012).

#### Fungal culture

The NM*Met*_SS9/2 and NM*Met*_CLPK4/1, which have been reported to be highly effective in controlling house fly larvae and pupae, respectively, were cultured on PDA for 14 days. Following this, a cork borer with a diameter of 0.7 mm was used to inoculate at the periphery of the colonised area, and they were then transferred to the centre of a Petri dish containing the medium. The experiment included five treatments: PDA alone, PDA + Cypas^®^ 250 EC at the recommended application rate (100 mL per 6.25 L), PDA + Cypas^®^ 250 EC at half the recommended application rate (50 mL per 6.25 L), PDA + Tanidil®-T at the recommended application rate (100 g/L ), and PDA + Tanidil®-T at half the recommended application rate (50 g/L). The experimental design followed a CRD, with each treatment replicated five times, and each replicate comprising five culture media plates.

The fungal culture was incubated in the dark at a temperature of 28°C–30°C and 70%–80%. R.H. The growth of fungus was measured by the colony diameter at intervals of 7 days, 14 days, 21 days and 28 days post-inoculation. The inhibition of mycelium growth (I) was computed using the following formula:


I (%)=[(Dc-Dt)/Dc]×100

where I (%) = inhibition percentage; Dc = average diameter of the control colonies and Dt = average diameter of the treated colonies.

### The Effectiveness of NM*Met*_SS9/2 and NM*Met*_CLPK4/1 Cultured on Media Containing an Insecticide in Controlling House Fly Larvae and Pupae

The 1 × 10^8^ spores/mL spore suspension of *Metarhizium* spp. isolates (NM*Met*_SS9/2 and NM*Met*_CLPK4/1) cultured on PDA and PDA mixed with Cypas^®^ 250 EC at half the recommended application rate were mixed with a semi-artificial diet in plastic cups containing house fly larvae or pupae. After inoculating the *Metarhizium* spp. isolates on larvae or pupae, they were transferred to an incubator in the dark at a temperature of 28°C–30°C and 60%–80% R.H. Daily observations of mortality and fungal infection were recorded for 14 consecutive days. Correction for mortality in the control treatment was done using Abbott’s formula (Abbott 1925). This experiment was compared with the fungus cultured on PDA, a control method without any insecticides, and Tween20^®^ (0.05%). The experiment was conducted using a CRD with three replicates for each method. Each replicate used 30 house fly larvae or pupae.

## DATA ANALYSIS

analysis was performed, which included an analysis of variance and a comparison of the mean differences among each treatment. This was conducted using Duncan’s New Multiple Range Test (DMRT) in conjunction with the Statistical Analysis System (SAS) software version 9.00 (SAS Institute Inc 2006).

## RESULTS

**Figure 1:**
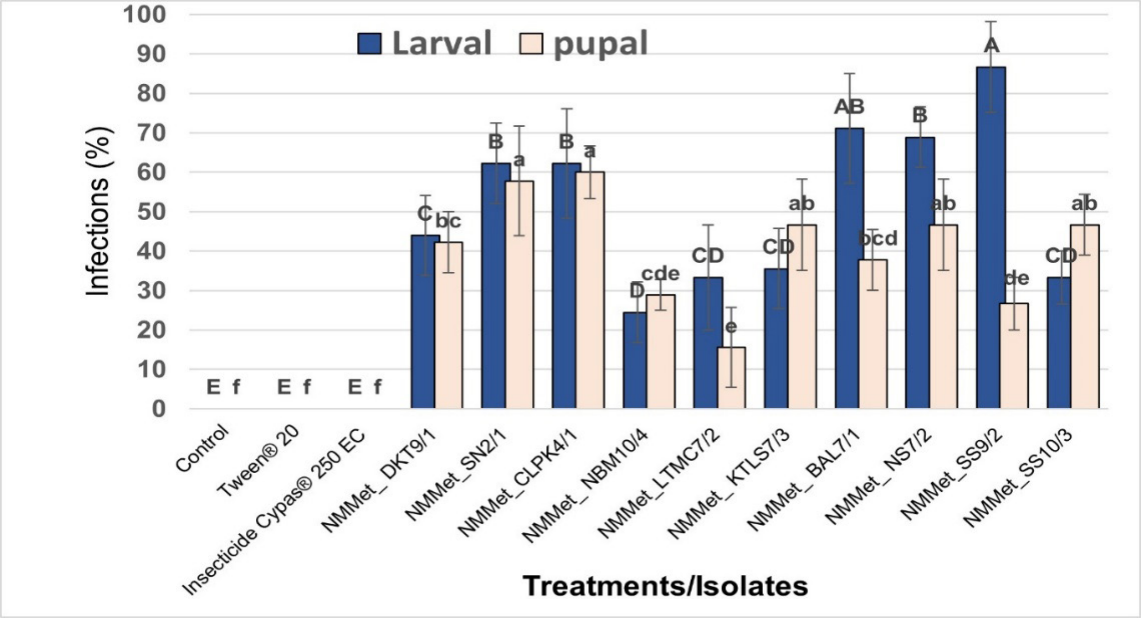
The effectiveness of *Metarhizium* spp. (1 × 10^8^ spores/mL) against 3rd instar larvae and pupae of the house fly (*Musca domestica* L.) was evaluated under laboratory conditions (28°C–30°C and 60%–80% R.H). Experiments were performed in triplicate. Error bars represent ± SD. Infection percentages followed by the same letter are not significantly different (*p* > 0.05) according to DMRT.

The 10 isolates of *Metarhizium* spp. were effective in controlling both larvae and pupae of house flies. For larvae control, NM*Met*_SS9/2 exhibited the highest infection percentage at 86.67%, but there was no statistical difference (*p* > 0.05) in the infection rates of NM*Met*_BAL7/1 (71.11%) isolate. Moreover, in the pupal test, NM*Met*_CLPK4/1 isolate showed the highest infection rate at 60.00%, which was statistically different (*p* < 0.05) from NM*Met*_LTMC7/2 isolate, which had the lowest infection rate at 15.56%. (Fig. 1).

### The Mycelial Growth of *Metarhizium* spp. on the Culture Medium Mixed with Insecticides

On the 28th day, both isolates, NM*Met*_SS 9/2 and NM*Met*_CLPK4/1 demonstrated superior growth on PDA mixed with Cypas^®^ 250 EC compared to PDA mixed with Tanidil^®^-T, as shown in Tables 2 and 3. The isolate NM*Met*_SS9/2 was unable to grow on PDA mixed with Tanidil^®^-T at the recommended application rate, exhibiting a significant mycelium growth inhibition of up to 93.60%. However, it was able to grow on PDA mixed with Tanidil^®^-T at half the recommended application rate, with a colony diameter of 20.83 mm and a mycelium growth inhibition of 74.99%. In the case of PDA mixed with Cypas^®^ 250 EC at a concentration of 50 mL per 6.25 L, the fungal colony diameters were 81.33 mm, showing no statistically significant differences (*p* < 0.05) in colony diameter compared to PDA alone (83.33 mm). At the recommended application rate (100 mL per 6.25 L) for PDA mixed with Cypas^®^ 250 EC, the fungal colony diameters were 49.00 mm, indicating a mycelium growth inhibition of 41.19%. Similarly, the isolate NM*Met*_CLPK4/1 cultured on PDA exhibited the largest colony diameter (80.50 mm), which was not significantly different (*p* > 0.05) from PDA mixed with Cypas^®^ 250 EC at half the recommended rate (77.67 mm), but was significantly different (*p* < 0.05) from other treatments. Conversely, when the fungus was cultivated on PDA combined with Tanidil^®^-T at both the recommended and half-recommended rates, it showed limited growth, with a colony diameter of 8.67 mm and 18.83 mm, respectively.

**Figure 2:**
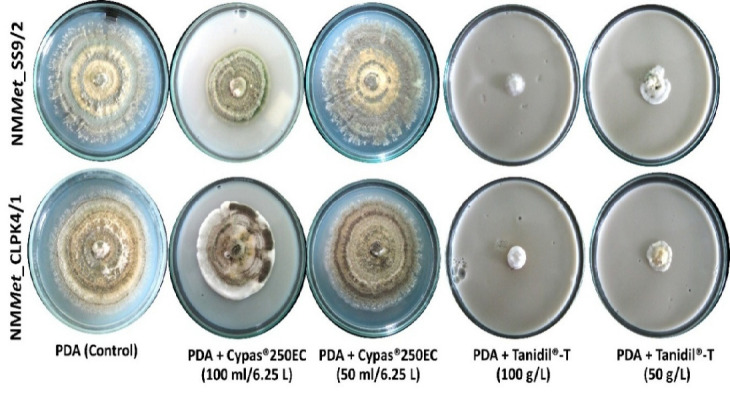
The colony characteristics of *Metarhizium* spp. isolates NM*Met*_SS9/2 and NM*Met*_CLPK4/1 on PDA mixed with insecticides—Cypas^®^ 250 EC (100 mL and 50 mL/6.25 L) and Tanidil^®^-T (100 g/L and 50 g/L)—were observed at 21 days.

Fig. 2 shows the characteristics of the fungal colony at 28 days on PDA and PDA mixed with Cypas^®^ 250 EC at half the recommended rate demonstrated that the colony and hyphae could smoothly proliferate on the agar surface. The colony on PDA initially presented as white with a slight green hue, transitioning to a green colour with a brownish tint as it matured. Conversely, when cultured on PDA mixed with Cypas^®^ 250 EC the hyphae exhibited slower growth, appearing white, with a dense compaction of hyphae around the colony edges. In the case of PDA mixed with Tanidil^®^-T, the hyphae failed to grow, and on PDA mixed with Tanidil^®^-T at half the recommended rate, the fungi produced only a limited number of white hyphae, predominantly clustered at the edge of the medium piece.

### The Effectiveness of the *Metarhizium* spp. Cultured on Media Mixed with an Insecticide in Controlling House Fly Larvae and Pupae

The NM*Met*_SS 9/2 isolate cultured on PDA mixed with Cypas^®^ 250 EC (50 mL per 6.25 L) demonstrated the highest mortality rate of house fly larvae at 93.33%. This rate was not statistically significantly different (*p* > 0.05) from the NM*Met*_SS 9/2 cultured on PDA alone (91.11%). The infection rates were closely comparable at 84.44% and 77.78% for the fungus cultured on PDA and PDA mixed with Cypas^®^, respectively. Similarly, the NM*Met*_CLPK4/1 isolate cultured on PDA mixed with Cypas^®^ 250 EC at half the recommended rate exhibited a mortality rate of 75.56% for house fly pupae. This rate was comparable to the NM*Met*_CLPK4/1 cultured on PDA alone (82.22%). The pupal infection rates were 73.33% and 71.11% for the fungus cultured on PDA and PDA mixed with Cypas^®^ 250 EC at half the recommended rate, respectively (Table 4).

## DISCUSSIONS

The results of this study indicated that all 10 isolates of *Metarhizium* spp. could infect both larvae and pupae of the house fly. However, the infection percentage varied among each isolate. Additionally, *Metarhizium* spp. could penetrate and destroy larvae of the house fly more rapidly than pupal stages. After a 2-day inoculation period, fungal hyphae were observed on the bodies of house fly larvae, whereas in the pupal stage, hyphae covered the pupae after 10 days of testing (data not shown in the table). This was consistent with Farooq and Feed (2016a), who reported that the virulence of insect destruction by fungi depends on the morphological characteristics such as age, sex and nutrition of the insect. Similarly, Sharififard *et al.* (2011a) reported that the LC_50_ values of *M. anisopliae* for controlling house flies differ depending on the life stage, with LC_50_ values ranging from 1.65 to 3 × 10^6^ conidia/g for adult stage and 7.3 × 10^4^ to 2.9 × 10^6^ conidia/mL for the larval stage. Additionally, Mishra *et al.* (2011) found that *M. anisopliae* is more effective in controlling house flies in the adult stage than in the larval stage. The time required for fungal penetration and destruction of insects, from spore contact to spore germination on the insect’s body, fungal growth within the insect and extrusion of hyphae, is approximately 96 h under favourable environmental conditions (temperature and humidity), but takes longer under unfavourable conditions (Mohammed 2018).

The ability of entomopathogenic fungi to control insects depends on several factors, such as the species and virulence of the entomopathogenic fungi, the target insect, the duration of entomopathogenic fungi contact with the insect, as well as environmental factors including temperature, humidity, sunlight and rainfall (Bugti *et al*. 2020; Quesada-Moraga *et al.* 2024). Some species of entomopathogenic fungi have specific host ranges. The destruction of insects by entomopathogenic fungi may be due to secondary metabolites or toxins produced by the fungi. However, some species of entomopathogenic fungi only invade and compete for essential mineral nutrients inside the insect’s body to sustain their own life (Bihal *et al.* 2023).

The compatibility of entomopathogenic fungi with insecticides test results revealed that Cypas^®^ 250 EC, a member of the pyrethroids class, exhibited less toxicity to the hyphal growth of *Metarhizium* spp. compared to Tanidil^®^-T. When Cypas^®^ 250 EC was applied at half the recommended rate (50 mL per 6.25 L of water) on both NM*Met*_SS9/2 and NM*Met*_CLPK4/1, the fungal hyphae could grow and produce spores. Conversely, when cultured on PDA mixed with Tanidil^®^-T, the fungi were unable to grow, with hyphal growth only occurring in the region where the fungus was deposited. This finding aligns with the results of Oliveira *et al.* (2003), who reported that Alpha-Cypermethrin, Thiamethoxam and Cyfluthrin exhibited lower inhibition to conidia germination at both field recommended and half-field recommended rates. Similarly, Oliveira *et al.* (2015) found that a Cypermethrin-6% wettable powder chemical insecticide, at recommended concentration, double recommended concentration and half recommended concentration, had no effect on the size of colony diameter but reduced spore viability and conidial production compared to the control (without insecticide). Apoorva and Ramaswamy (2013) conducted compatibility testing of *Metarhizium anisopliae* with three organophosphate compounds (phorate, malathion and chlorpyrifos) and two pyrethroids (deltamethrin and permethrin), finding that phorate was notably more toxic to *M. anisopliae* than the other pesticides, significantly inhibiting both vegetative growth and sporulation. Similarly, Schumacher and Poehling (2012) examined the *in vitro* effects of various pesticide concentrations, including fipronil, imidacloprid, neemazal and amitraz, on *M. anisopliae*. Their study revealed that only fipronil, an organophosphate, exhibited moderate toxicity to *M. anisopliae* at a concentration of 200 ppm.

The compatibility between insecticides and entomopathogens depends on various factors. For instance, Tamai *et al.* (2002) reported that insecticide products with similar modes of action, produced by different companies, may elicit different responses from pathogens due to variations in the ingredients (inert ingredients and adjuvants) used in each product’s formulation. Such variations are contingent on the presence of compounds that block conidia metabolic functions and the concentrations of active compounds (Antonio *et al.* 2001; Kumar *et al.* 2000).

The mechanism of action of insecticides on entomopathogens is described by Oliveira *et al.* (2003). They reported that molecules analogous to prosthetic groups diffuse to the cytoplasm, where they bind to specific receptors, affecting membrane permeability and enzymatic synthesis. Consequently, this process influences metabolic processes, and the same inhibitory mechanism is likely responsible for differences in conidial germination and vegetative growth in *M. anisopliae*. Additionally, the interaction depends on the type and isolate of the fungus, as different isolates of entomopathogenic fungi exhibit varying levels of tolerance to insecticides. Furthermore, these fungi can also degrade the pyrethroid cypermethrin (Oliveira *et al.* 2016).

NM*Met*_SS9/2 and NM*Met*_CLPK4/1 cultured on PDA mixed with Cypas® 250 EC at half the recommended rate were used in controlling both larvae and pupae of house flies. Culturing on PDA mixed with Cypas^®^ 250 EC did not affect the efficacy of insect pest control. These findings align with the results of Jantanapim *et al.* (2015), who reported that *B. bassiana* cultured on PDA combined with Thiamethoxam resulted in the highest mortality of cassava mealybug, with no statistical significance (*p* > 0.05) compared to the fungus cultured on PDA. Similarly, Ponganan *et al.* (2015) reported that *B. bassiana* cultured on PDA combined with Buprofezin caused the highest mortality of brown planthoppers, with no significant difference compared to the fungus cultured on PDA. This may be attributed to the ability of entomopathogenic fungi to degrade insecticides. Oliveira *et al.* (2016) reported that the fungus *B. bassiana* can degrade Cypermethrin. In a similar vein, Ong *et al.* (2019) found that *M. anisopliae* can degrade both insecticides (Chlorpyrifos and Cypermethrin) by more than 80%, which is significantly higher than the control soil (47%–61%). Abd El-Ghany and Masmali (2016) demonstrated that *M. anisopliae* can degrade Organophosphorus by more than 90%, sharing the same category and action mode as chlorpyrifos.

In addition to using entomopathogenic fungi cultured on media containing insecticides for pest control, there is also a trend of using these Entomopathogenic fungi in combination with insecticides to control various insect species. The effectiveness of insect control does not decrease when fungi are combined with insecticides. Sharififard *et al.* (2011b) combined *M. anisopliae* with Spinosad to control house flies, resulting in a synergistic effect that increased house fly mortality and reduced the lethal time. Similarly, Ong *et al.* (2017) found that the combination of *M. anisopliae* and a mixture of Chlorpyrifos and Cypermethrin (ChCy) resulted in 62%–72% house fly larva mortality. Furthermore, Farooq and Freed (2016b) used *B. bassiana* in combination with insecticides to control house flies. They discovered that when *B. bassiana* was combined with acetamiprid, Emamectin, Imidacloprid or lufenuron, there was a significant decrease in longevity, fecundity, egg hatching, percent pupation, pupal weight and adult emergence. Additionally, larval duration and pupal period were prolonged.

## CONCLUSION

The NM*Met*_SS9/2 and NM*Met*_CLPK4/1 isolates were identified as the most effective in controlling house fly larvae and pupae, respectively. Both isolates exhibited enhanced growth on PDA supplemented with Cypas^®^ 250 EC at a concentration of 50 mL per 6.25 L, compared to PDA mixed with Tanidil®-T, which significantly inhibited fungal growth. Spore suspensions obtained from cultures grown on PDA supplemented with Cypas^®^250 EC demonstrated insect pest control effectiveness comparable to those cultured on PDA alone, with no statistically significant differences observed. This study validates the potential of Cypermethrin, a synthetic pyrethroid, to be effectively combined with the fungus *Metarhizium* spp. for controlling house flies. This approach establishes a foundation for utilising Cypermethrin in conjunction with other microorganisms to manage various insect species in the future.

## Figures and Tables

**Table 1:** Chemical insecticides, active ingredients and recommended application rates.

Commercial name	Ingredient	Chemical class	Recommended application rate	Manufacturer
Cypas^®^ 250 EC	Cypermethrin 25%	Pyrethroids	100 mL/6.25 L	Q Fac Company Limited
Tanidil^®^-T	Coumaphos 3%; Propoxur 2%	Organophosphate	100 g/1 L	Bayer

**Table 2:** Colony diameter and mycelial growth inhibition of the *Metarhizium* spp. isolate NM*Met*_SS9/2 on culture medium mixed with insecticides.

Treatments	Parameters1/							

	Colony diameter (mm ± SD)				Mycelium growth inhibition over control (% ± SD)			

	7 days	14 days	21 days	28 days	7 days	14 days	21 days	28 days
PDA (Control)	28.83 ± 1.04^a^	46.00 ± 2.60^a^	70.17 ± 1.89^a^	83.33 ± 0.76^a^	0.00 ± 0.00^d^	-	-	-
PDA+Cypas^®^ 250 EC.2/	20.33 ± 1.53^b^	30.67 ± 0.76^c^	42.33 ± 2.84^c^	49.00 ± 1.32^b^	29.54 ± 2.75^c^	33.18 ± 4.30^c^	39.56 ± 5.75^c^	41.19 ± 2.13^ab^
PDA+Cypas^®^ 250 EC. (½)3/	17.17 ± 2.02^c^	35.00 ± 1.32^b^	65.00 ± 1.73^b^	81.33 ± 1.04^a^	40.29 ± 8.72^b^	23.79 ± 4.30^d^	7.34 ± 2.73^d^	2.40 ± 0.60^c^
PDA+Tanidil^®^-T4/	5.00 ± 0.00^d^	5.00 ± 0.00^e^	5.00 ± 0.00^e^	5.33 ± 0.29^e^	82.64 ± 0.62^a^	89.11 ± 0.60^a^	92.87 ± 0.20^a^	93.60 ± 0.39^a^
PDA+Tanidil^®^-T(½)5/	5.00 ± 0.00^d^	12.17 ± 1.89^d^	15.67 ± 0.58^d^	20.83 ± 1.61^d^	82.64 ± 0.62^a^	73.55 ± 3.93^b^	77.65 ± 1.29^b^	74.99 ± 2.12^a^
*p-*value	< 0.0001	< 0.0001	< 0.0001	< 0.0001	< 0.0001	< 0.0001	< 0.0001	0.0104

*Notes*.

1/ Means ± SD within a column followed by the same letter are not significantly different ( DMRT, *p* > 0.05), SD = standard deviation;

2/ Cypas^®^ 250 EC 100 mL/6.25 L ( recommended application rate);

3/ Cypas^®^ 250 EC 50 mL/6.25 L ( half-recommended application rate);

4/ Tanidil^®^-T 100 mL/1 L ( recommended application rate);

5/ Tanidil^®^-T 50 mL/1 L ( half-recommended application rate).

**Table 3:** Colony diameter and mycelial growth inhibition of the *Metarhizium* spp. isolate NM*Met*_CLPK4/1 on culture medium mixed with insecticides.

Treatments	Parameters1/							

	Colony diameter (mm ± SD)				Mycelium growth inhibition over control (% ± SD)			

7 days	14 days	21 days	28 days	7 days	14 days	21 days	28 days
PDA (Control)	28.00 ± 0.05^a^	46.67 ± 1.89^a^	65.83 ± 2.47^a^	80.50 ± 3.61^a^	0.00 ± 0.00^d^	-	-	-
PDA+Cypas^®^ 250 EC2/	19.00 ± 0.05^c^	31.17 ± 0.29^c^	46.83 ± 0.29^c^	59.00 ± 1.80^b^	32.14 ± 1.57^b^	33.15 ± 2.44^c^	28.80 ± 2.28^c^	26.55 ± 5.42^c^
PDA+Cypas^®^ 250 EC(½)3/	21.83 ± 1.04^b^	37.76 ± 1.15^b^	56.00 ± 0.50^b^	77.67 ± 1.04^a^	21.98 ± 4.86^c^	19.22 ± 3.24^d^	14.84 ± 3.92^d^	3.45 ± 2.36^d^
PDA+_Tanidil_^®^-T4/	5.00 ± 0.00^d^	5.00 ± 0.00^e^	6.00 ± 0.00^e^	8.67 ± 0.76^d^	82.14 ± 0.32^a^	89.27 ± 0.44^a^	90.88 ± 0.35^a^	89.19 ± 1.46^a^
PDA+Tanidil^®^-T(½)5/	5.00 ± 0.00^d^	10.50 ± 0.87^d^	11.67 ± 2.89^d^	18.83 ± 1.04^c^	82.14 ± 0.32^a^	77.51 ± 1.45^b^	82.33 ± 3.97^b^	76.54 ± 2.32^b^
*p-*value	< 0.0001	< 0.0001	< 0.0001	< 0.0001	< 0.0001	< 0.0001	< 0.0001	< 0.0001

*Notes.*

1/ Means ± SD within a column followed by the same letter are not significantly different ( DMRT, *p* > 0.05), SD = standard deviation;

2/ Cypas^®^ 250 EC 100 mL/6.25 L ( recommended application rate);

3/ Cypas^®^ 250 EC 50 mL/6.25 L (half-recommended application rate);

4/ Tanidil^®^-T 100 mL/1 L ( recommended application rate);

5/ Tanidil^®^-T 50 mL/1 L ( half-recommended application rate).

**Table 4:** The effectiveness of the *Metarhizium* spp., isolates NM*Met*_SS9/2 and NM*Met*_CLPK4/1, cultured on culture medium mixed with insecticide, in controlling house fly (*Musca domestica* L.).

Treatments	NM*Met*_SS 9/2		NM*Met*_CLPK 4/1	

	Larval mortality (% ± SD)	Larval infection (% ± SD)	Pupal mortality (% ± SD)	Pupal infection (% ± D)
Control (non-spraying)	4.44 ± 3.85^b^	0.00 ± 0.00^b^	0.00 ± 0.00^b^	0.00 ± 0.00^b^
Tween^®^ 20 0.05%	8.89 ± 3.85^b^	0.00 ± 0.00^b^	0.00 ± 0.00^b^	0.00 ± 0.00^b^
Fungus cultured on PDA	91.11 ± 3.85^a^	84.44 ± 10.18^a^	82.22 ± 7.70^a^	73.33 ± 11.55^a^
Fungus cultured on PDA+Cypas^®^ 250 EC1/	93.33 ± 6.67^a^	77.78 ± 3.85^a^	75.56 ± 3.85^a^	71.11 ± 3.85^a^
*p-*value	< 0.0001	< 0.0001	< 0.0001	< 0.0001

*Notes*. Means ± SD within a column followed by the same letter are not significantly different (DMRT, *p* > 0. 05); SD = standard deviation;

1/ Cypas^®^ 250 EC 50 mL/6.25 L of water (half recommended application rate).
